# Reversibility of Central Nervous System Adverse Events in Course of Art

**DOI:** 10.3390/v14051028

**Published:** 2022-05-11

**Authors:** Lucia Taramasso, Giancarlo Orofino, Elena Ricci, Barbara Menzaghi, Giuseppe Vittorio De Socio, Nicola Squillace, Giordano Madeddu, Francesca Vichi, Benedetto Maurizio Celesia, Chiara Molteni, Federico Conti, Filippo Del Puente, Eleonora Sarchi, Goffredo Angioni, Antonio Cascio, Carmela Grosso, Giustino Parruti, Antonio Di Biagio, Paolo Bonfanti

**Affiliations:** 1 Infectious Disease Clinic, IRCCS Policlinico San Martino Hospital, 16132 Genoa, Italy; 2 Unit of Infectious Diseases, “Divisione A”, Amedeo di Savoia Hospital, 10149 Torino, Italy; giancarlo.orofino@aslcittaditorino.it; 3 Fondazione ASIA Onlus, 20090 Buccinasco, Italy; ed.ricci@libero.it; 4 Unit of Infectious Diseases, ASST della Valle Olona, Busto Arsizio Hospital, 21052 Busto Arsizio, Italy; barbara.menzaghi@asst-valleolona.it; 5 Clinic of Infectious Diseases, Department of Medicine, Azienda Ospedaliera di Perugia, Santa Maria Hospital, 06129 Perugia, Italy; giuseppedesocio@yahoo.it; 6 Infectious Diseases Clinic, San Gerardo Hospital, University of Milano-Bicocca, 20126 Monza, Italy; n.squillace@asst-monza.it (N.S.); paolo.bonfanti@unimib.it (P.B.); 7 Unit of Infectious and Tropical Diseases, Department of Medical, Surgical and Experimental Sciences, University of Sassari, 07100 Sassari, Italy; giordano@uniss.it; 8 Infectious Diseases Unit 1, Santa Maria Annunziata Hospital, Azienda USL Toscana Centro, 50012 Florence, Italy; francesca.vichi@uslcentro.toscana.it; 9 Unit of Infectious Diseases, University of Catania, ARNAS Garibaldi, 95123 Catania, Italy; bmcelesia@tin.it; 10 Infectious Diseases Unit, Ospedale A. Manzoni, 23900 Lecco, Italy; c.molteni@asst-lecco.it; 11 Infectious Diseases Unit, Department of Biomedical and Clinical Sciences “Luigi Sacco”, Università Degli Studi di Milano, 20122 Milan, Italy; federico.conti@unimi.it; 12 Department of Health Sciences, Infectious Disease Clinic, University of Genoa, 16145 Genoa, Italy; fildelp@gmail.com (F.D.P.); antonio.dibiagio@hsanmartino.it (A.D.B.); 13 Infectious Diseases Unit, SS. Antonio e Biagio e Cesare Arrigo Hospital, 15121 Alessandria, Italy; eleonora.sarchi@ospedale.al.it; 14 Infectious Diseases Unit, SS Trinità Hospital, 09121 Cagliari, Italy; goffredoangioni@gmail.com; 15 Infectious and Tropical Diseases Unit, Department of Health Promotion, Mother and Child Care, Internal Medicine and Medical Specialties (PROMISE), University of Palermo, 90133 Palermo, Italy; antonio.cascio03@unipa.it; 16 Unit of Infectious Diseases, Cesena Hospital, 47521 Cesena, Italy; c.grosso1@virgilio.it; 17 Infectious Diseases Unit, Pescara General Hospital, 66020 Pescara, Italy; parrutig@gmail.com

**Keywords:** CNS, adverse events, HIV, dolutegravir, reversibility, neurocognitive, psychiatric

## Abstract

The purpose of this study is to evaluate the frequency of central nervous system adverse events (CNS-AE) on dolutegravir (DTG) and non-DTG containing ART, and their reversibility, in the observational prospective SCOLTA cohort. Factors associated with CNS-AE were estimated using a Cox proportional-hazards model. 4939 people living with HIV (PLWH) were enrolled in DTG (*n* = 1179) and non-DTG (*n* = 3760) cohorts. Sixty-six SNC-AE leading to ART discontinuation were reported, 39/1179 (3.3%) in DTG and 27/3760 (0.7%) in non-DTG cohort. PLWH naïve to ART, with higher CD4 + T count and with psychiatric disorders were more likely to develop a CNS-AE. The risk was lower in non-DTG than DTG-cohort (aHR 0.33, 95% CI 0.19–0.55, *p* < 0.0001). One-year follow-up was available for 63/66 PLWH with CNS-AE. AE resolution was reported in 35/39 and 23/24 cases in DTG and non-DTG cohorts, respectively. The probability of AE reversibility was not different based on ART class, sex, ethnicity, CDC stage, or baseline psychiatric disorder. At the same time, a lower rate of event resolution was found in PLWH older than 50 years (*p* = 0.017). In conclusion, CNS-AE leading to ART discontinuation was more frequent in DTG than non-DTG treated PLWH. Most CNS-AE resolved after ART switch, similarly in both DTG and non-DTG cohorts.

## 1. Introduction

In the modern antiretroviral therapy (ART) era, the goals of successful treatment should go beyond virological success and CD4 + T cell count restoration and include the tolerability and treatment satisfaction of people living with HIV (PLWH) [1]. To guarantee all these factors, the screening and management of the undesired Central Nervous System (CNS) adverse events (AEs) which occur during ART is pivotal, and bringing them to zero can be particularly important for improving the quality of life of PLWH. However, CNS-AEs might often be underdiagnosed [2]. Additionally, little is known about which percentage of events resolve completely after ART switches and if an inter-class switch could be associates with a different outcome than an intra-class switch. The picture can be further complicated by difficulties in distinguishing between an ART-induced CNS side effect and symptoms like anxiety, depression, or sleep disturbance that can also be caused by the psychological impact of a new HIV diagnosis in PLWH undergoing their first-line ART. Moreover, organic causes of cognitive impairment can also be present, such as pre-existing neurological or psychiatric comorbidities, CNS opportunistic diseases, or neurologic manifestations of the HIV-1 infection itself [3]. On the other side, ART-related CNS-AEs may also be present and have been described, with variable frequency, across all antiretroviral classes. Among non-nucleoside reverse transcriptase inhibitors (NNRTI), efavirenz is the one with the highest incidence of CNS-AEs [4]. At the same time, the two other NNRTI that remained in use in recent ART, rilpivirine (RPV) and doravirine (DOR), although not totally free from CNS AEs of various degrees, are characterized by better CNS tolerability [5,6,7,8]. Among protease inhibitors (PIs), darunavir (DRV) is the only one still counselled by current guidelines [9,10]. Additionally, for this drug, CNS-AEs such as headaches, insomnia, dizziness, depression, or anxiety have been described as varying between 1 and 15%, although these are not always drug-related and rarely leading to drug discontinuation [11,12,13]. Finally, among integrase inhibitors (INSTIs), dolutegravir (DTG) is one of the preferred agents in first-line ART and switch strategies. However, observational data highlighted a certain rate of neurological and psychiatric disorders occurring in the course of treatment [14], owing to drug discontinuation in the variable frequency of PLWH [15,16,17,18,19,20], up to a maximum of 9.9% in a single Dutch study [21]. For other INSTIs such as raltegravir (RAL) and elvitegravir (EVG), the incidence of CNS toxicity reports seems lower in the literature [22,23]. At the same time, for bictegravir (BIC), observational data are still scarce [24], but for all of them, CNS-AEs have been signaled in the literature with variable incidence [24,25,26,27].

The aim of the present study was to evaluate the frequency and risk factors for CNS side effects leading to drug discontinuation in DTG and non-DTG treated PLWH and to describe the frequency of CNS AE resolution after an ART switch.

## 2. Materials and Methods

### 2.1. Study Population

We analyzed data from the SCOLTA (Surveillance Cohort Long-Term Toxicity Antiretrovirals) prospective database. The SCOLTA project is a multicenter observational study started in 2002. It follows prospective PLWH who start to take new antiretroviral drugs, to identify toxicities and AEs in a real-life setting [28]. The SCOLTA project uses an online pharmacovigilance program and involves 25 Italian Infectious Disease Centers [28].

Both ART naïve and experienced patients can be included in SCOLTA if they are >18 years and agree to enter the study. Clinical data collected include sex, age, ethnicity, weight, height, CDC stage, and previous ART history. Laboratory data include HIV-RNA, CD4 + T cell count, and biochemical data. The data was prospectively collected in anonymous form in a central database every six months. AEs are collected as soon as they are clinically observed. CNS-AEs included: altered mental status, cognitive, behavioral, or attentional disturbances, sleep disturbances, headaches, seizures, and painful neuropathy. The AE was considered severe (grade 3 or 4 AE) in the case of serious changes in behavior/humor requiring medical intervention or in cases of acute psychosis requiring hospitalization. For the present study, all AEs affecting the CNS that led to the discontinuation of the study drug were evaluated for PLWH initiating lopinavir/ritonavir (LPV/r, *n* = 731), atazanavir/ritonavir (ATV/r, *n* = 616), DRV/ritonavir or DRV/cobicistat (DRV/r or DRV/c) (*n* = 721), RPV (*n* = 481), RAL (*n* = 514), EVG (*n* = 339), BIC (*n* = 358) or DTG (*n* = 1179) in the observational SCOLTA study. Only PLWH with at least one follow-up visit after enrollment in SCOLTA were considered eligible. The time in which study participants were enrolled depended on the period in which enrolment was opened for each of the study cohorts, which was as follows: LPV/r October 2002–November 2004 [28]; ATV/r January 2003–May 2008 [29]; DRV/r May 2006–August 2012 [30]; DRV/c April 2016–September 2018 [31]; RPV January 2013–September 2017 [8]; RAL October 2007–June 2014 [32]; EVG January 2014–October 2017 [33]; BIC July 2019, ongoing: DTG July 2014, ongoing [20,34].

For each CNS-AE, the reversibility of the event was checked one year after the ART switch was performed. Complete data collection and follow-up procedures for the cohorts have been previously described [28,35].

The original study protocol was approved on 18 September 2002. A new protocol amendment was approved on 13 June 2013 by the coordinating center at Hospital “L. Sacco”-University of Milan, Milan (Italy) and thereafter by all participating centers. Written consent for study participation was obtained from all participants. The study was conducted in accordance with the ethical standards laid down in the 1964 Declaration of Helsinki and its later amendments and by Italian national laws.

### 2.2. Objectives

The primary study objective was to evaluate the frequency of CNS-AEs leading to ART discontinuation in DTG and non-DTG cohorts and their reversibility after switching ART.

The secondary objective was the evaluation of factors associated with CNS-AEs occurrence in all study participants, in those on DTG containing ART and those in non-DTG containing ART.

### 2.3. Statistical Analysis

Data were described using mean and standard deviation (SD) for normally distributed continuous variables, median, and interquartile range (IQR) for not normally distributed continuous variables and frequency (%) for categorical and ordinal variables. The rates of treatment discontinuations due to CNS-AEs were calculated as the number of discontinuations (95% confidence interval, CI) per 100 patient-years follow-up (PYFU), and the Rate Ratio (95% CI) was estimated using the Mid-P exact method. Frequencies of CNS-AE related discontinuations in DTG and non-DTG cohorts were compared using Kaplan-Meyer survival curves (Log-Rank test).

The frequency of CNS-AEs reversibility was compared across different characteristics of study participants using a Chi-Squared test or Fisher’s exact test, as appropriate.

The Cox proportional-hazards model was used for estimating the hazard ratio (HR) and 95% CI of CNS-AEs leading to ART discontinuations in all the study population, DTG, and non-DTG-cohorts.

For patients at risk of ART discontinuation due to CNS-AEs as well as due to other reasons (other AEs, treatment failure, clinical events), we generated a competing risk model accounting for competing risk of discontinuation due to any other reasons than CNS-AEs.

All p-levels were two-sided, at the significance level of < 0.05. All statistical analyses were performed using SAS for Windows 9.4 (SAS Institute, Cary, NC, USA).

## 3. Results

PLWH enrolled were 4939, 1179 in DTG and 3760 in non-DTG cohorts. Among them, 1334 (27.0%) were female, mean age was 45.2 (SD 12.6) years old, and median CD4 + T cell count was 410 (IQR 215—660) cells/mmc. Eight hundred and thirty-four PLWH (16.9%) were ART naïve, while, among the remaining 4,105 ART-experienced PLWH, 2289 (55.8%) had HIV-RNA *<* 50 copies/mL when they started the study drug. Most PLWH were in CDC stage A (*n* *=* 2085, 42.2%), while others were similarly distributed between stages B (*n* *=* 1414, 28.6%) and C (*n* *=* 1440, 29.2%). DTG-treated PLWH were more often male, non-Caucasian, and were, on average older, with a less advanced stage of HIV infection, higher rate of viral suppression (HIV-RNA *<* 50 copies/mL), and lower frequency of HCV co-infection, when compared to the non-DTG cohort. Meanwhile, at baseline, no significant difference existed in the frequency of neuropsychiatric disorders in the two cohorts (Table 1). A baseline psychiatric disorder was diagnosed in 3.4% of ART-naïve and 7.1% of ART-experienced PLWH across the study cohorts. The median observation time was 23 months (IQR 12–39).

### 3.1. CNS-AEs Leading to ART Discontinuation

**Figure 1:**
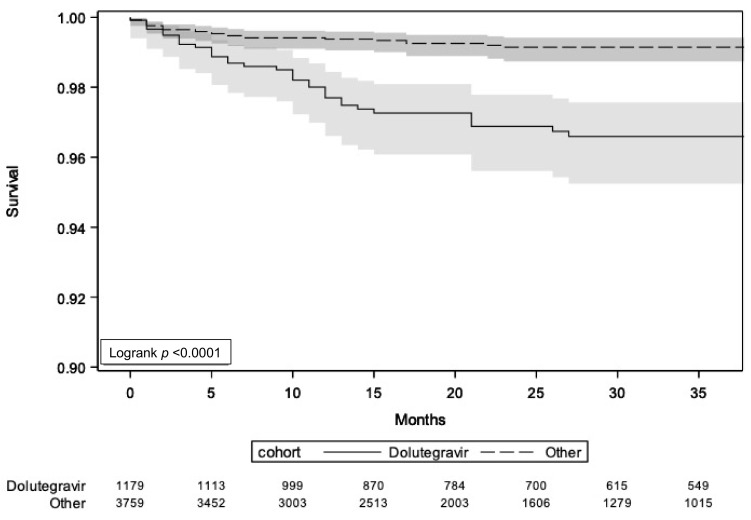
Survival analysis in strata of DTG and non-DTG-based regimens showed a significantly higher rate of discontinuations due to CNS-AE in DTG (logrank *p* < 0.0001).

Sixty-six CNS-AEs leading to ART discontinuation were reported, of which 21/66 (32%) were considered non-severe and 32/66 (48%) severe, while for 13 events (20%), the grade of severity was not reported. Among the 66 events, 27/3760 were in non-DTG-cohorts (2/731 LPV/r, 1/616 ATV/r, 2/721 DRV/r or DRV/c, 8/481 RPV, 5/514 RAL, 3/339 EVG, and 6/358 BIC) and 39/1179 were in DTG-cohort, with an estimated discontinuation rate of 0.32 (95% CI 0.19–0.52) per 100 PYFU in non-DTG and 1.14 (95% CI 0.81–1.56) per 100 PYFU in DTG cohort, with a rate ratio: 0.32 (95% CI 0.19–0.52). Survival analysis also showed a significant difference between groups in terms of discontinuations due to CNS-AEs (logrank *p* *<* 0.0001, Figure 1).

A longer time between drug initiation and CNS-AE was observed in the DTG group than in the non-DTG group (10.0 (IQR 4–21) versus 4.0 (IQR 1–7) months (*p* *=* 0.005)).

Eight PLWH in DTG and six in non-DTG reported more than one CNS-AE. The CNS-AEs reported were, (frequency in DTG and in non-DTG cohorts): sleep disturbance (*n* *=* 13 and 6); agitation (*n* *=* 4 and 3); depression (*n* *=* 6 and 3); headache (*n* *=* 7 and 7); vertigo (*n* *=* 5 and 4); suicidal ideation (*n* *=* 3 and 1); psychosis (*n* *=* 1 and 2); anxiety (*n* *=* 2 and 0); other CNS disturbances (*n* *=* 4 and 4).

### 3.2. Reversibility after Switching ART

After drug discontinuation, a one-year follow up was available for 63/66 PLWH experiencing CNS-AEs, 39 in DTG and 24 in non-DTG cohort. Among them, 26 (18 DTG and 8 non-DTG) were switched to an INSTI, 14 (9 DTG and 5 non-DTG) to NNRTI, and 24 to PI (12 DTG and 12 non-DTG). Of them, two were switched to INSTI+ NNRTI and one to NNRTI+ PI. Two PLWH discontinued ART by their own choice against medical advice.

The frequency of AE resolution was similar in DTG and non-DTG cohorts, and PLWH who were switched to a different ART class (inter-class switch) did not show different AE outcomes compared to those switching to another drug of the same ART class (intra-class switch), with AE resolution in 36/37 inter-class and in 22/26 intra-class switches (Table 2). Also, the probability of AE reversibility was not different based on sex, ethnicity, CD4 + T-cell count, or CDC stage, nor on people with or without a previous diagnosis of a psychiatric disorder, while older PLWH were those less likely to resolve the CNS-AE in this cohort (*p* *=* 0.017, Table 2).

Five PLWH still reported CNS-AE persistence one-year after drug discontinuation (four discontinuing DTG and one RPV): one case of worsening symptoms of pre-existing diagnosis of depression, one case of worsening symptoms of a pre-existing diagnosis of psychosis, two cases of anxiety/agitation, and one case of neuropathy/neuropathic pain.

### 3.3. Factors Associated with CNS-AEs

In the whole study population, after adjusting for possible confounders (Table 3), PLWH naïve to ART (adjusted Hazard Ratio, aHR 2.23, 95% CI 1.24–3.99, *p* *=* 0.007) and those with a previous diagnosis of psychiatric disorder (aHR 2.13, 95% CI 1.04–4.34, *p* *=* 0.038) were more likely to develop a CNS-AE. Moreover, the risk of CNS-AE was lower in PLWH who did not receive DTG compared to DTG-cohort (aHR, 0.33, 95% CI, 0.19–0.55, *p* *<* 0.0001). Adjusted HR for each single drug compared to DTG and comparison between INSTI and non-INSTI cohorts are available in Supplementary Materials.

When repeating the analysis in strata of the study cohort (DTG or non-DTG), being naïve to ART was confirmed to be a significant risk factor for CNS-AE only in DTG treated PLWH (HR 2.53, 95% CI 1.35–4.73, *p* *=* 0.004). However, in the non-DTG cohort, the presence of a pre-existing diagnosis of psychiatric disorder (HR 4.15, 95% CI 1.67–10.33, *p* *=* 0.002) and higher levels of CD4 + T-cell counts (≥750 cells/mmc, compared to <250 cells/mmc, HR 40.4, 95% CI 1.21–13.44, *p* *=* 0.02), mutually adjusted, resulted in predictors of CNS-AE (Table 4).

## 4. Discussion

In this observational prospective study cohort, we found a higher incidence of CNS-AE in DTG compared to non-DTG-treated PLWH. However, most AEs were reversible and resolved after an ART switch, either to a drug of the same class or to other drug classes, without significant differences in the probability of AE resolution between intra-class or inter-class switches. We found an incidence of CNS-AE leading to DTG discontinuation of about 1.1 per 100 PYFU (3.3% of DTG-treated PLWH in the study). This frequency was lower when compared to some previous reports, where DTG discontinuations due to neuropsychiatric toxicity ranged between 5.2 and 9.9% [17,21,27,36], but similar to that reported in other European cohorts [23,25,37] and still higher than that described in clinical trials, where on average 2% of all the study participants discontinued DTG due to any AE, including also CNS-AEs [38,39,40,41,42,43]. There could be multiple reasons for this variable incidence, depending on different study populations, the prevalence of advanced HIV stages, comorbidities, and complex therapies taken used concurrently with DTG in different observational studies, often including PLWH that do not meet the criteria for inclusion in clinical trials.

When analyzing which factors were associated with CNS-AEs, we found a discordant result, namely that they were more frequent in naive PLWH but also those with higher CD4 + T cell counts. However, stratifying the analysis by cohort, separating DTG and non-DTG regimens, showed that risk factors for CNS-AEs were not the same in the two groups. For PLWH on DTG, being ART naive resulted in the strongest (and the only statistically significant) predictor of AE. Many factors may have played a role in this finding and contributed to the emergence of CNS-AEs, including the psychological impact and personal concerns of people newly diagnosed with HIV infection, which may result in a high level of the burden associated with a recent diagnosis [44], but also the possibility of opportunistic diseases and IRIS, and, not least, the neurological manifestations of the untreated HIV-1 infection itself [3,45]. Instead, the prevalence of psychiatric disorders at baseline was lower in ART-naïve than in ART-experienced PLWH, so this factor did not seem to have influenced the outcome unless some psychiatric comorbidities were still undiagnosed at the time of study enrollment in people recently diagnosed with HIV [46].

In contrast, in the non-DTG cohort, PLWH with higher CD4 + T counts and those with baseline psychiatric disorders were more at risk of CNS-AEs. In this group, it seemed that symptoms were not mediated by possible infectious complications due to low CD4 + T counts. On the contrary, PLWH with high CD4 + T, in the presence of psychiatric comorbidity, were those at higher risk. The other factors examined, in the whole group and in the stratified analysis, did not show a consistent association with the outcome. However, age showed a trend towards association with AEs, in accordance with previous studies which found higher rates of CNS-AE in older PLWH and that hypothesized a possible impact of higher drug concentrations in such context, based on a possible but debated correlation between drug exposure and AE probability [27,47,48,49,50,51,52,53].

The reasons why the risk factors for AEs should be different in DTG and non-DTG cohorts remain unclear. Many mechanisms have been advocated for in past years to explain the CNS-AEs in the course of DTG, including higher CNS concentration of drugs [47,48,50,52,53], interactions with SLC22A2 gene expression [51], mitochondrial dysfunction, and the alterations in HeLa Epithelial and BV2 Microglial Cells [54]. Additionally, for other antiretroviral drugs and drug classes, there are several possible mechanisms. For instance, among NNRTIs, the most studied is EFV, whose CNS toxicity has been linked to increased pro-inflammatory cytokines such as tumor necrosis factor alpha and interleukin-1β, reduced creatine kinase levels in the brain, mitochondrial toxicity, autophagy, and endoplasmic reticulum stress, with higher effects at higher drug concentrations [55]. Regarding PIs, several studies have indicated they could potentiate neuropathy associated with NRTIs, with neurotoxicity mostly pronounced in combination treatments as compared to individual PIs [55]. However, the actual pathogenesis of drug-incident CNS-AEs remains still to be fully understood and is probably multifactorial. In our study, the two cohorts considered had different baseline characteristics, which could have influenced the final observations. PLWH enrolled in DTG were generally older, with less advanced HIV disease and more frequently ART naïve and, importantly, enrolled in more recent years due to the year of commercialization of DTG in Italy. This could have brought a higher awareness towards CNS-AEs in general and might also have conditioned a higher number of toxicity reports compared to past years and older drugs, also in the light of other reports of DTG-incident CNS-AEs [23]. However, although the actual mechanisms of neuropsychiatric toxicity remain the object of studies, all the hypothesized mechanisms seem reversible at the time of drug withdrawal. In line with these hypotheses, almost all CNS-AEs reported in the present study resolved after a one-year follow-up. Moreover, none of the features that we examined seemed predictive of CNS-AE non-resolution, except for older age, which might also be interpreted as an independent risk factor for neurocognitive disorders in ageing PLWH independently from ART exposure [56,57,58]. The lack of recovery of neuropsychiatric symptoms after discontinuation of the drug, however, might also be interpreted as a lack of an actual correlation with the drug rather than as a sign of persisting damage. On the other hand, all resolutions happened immediately after discontinuation of ART, suggesting that, at least in this study, most drug-induced CNS-AEs did not involve long-term effects after discontinuation.

The present study had several limitations. These include its observational, non-randomized design and the lack of a specific neuropsychiatric evaluation to objectivate the symptoms and their severity before and after drug discontinuations. Additionally, a significant limitation is the different years in which PLWH treated with different drugs were enrolled in the study, covering quite a long period, reflecting different ART eras and knowledge across years. The study of PLWH with different baseline characteristics is considered another limitation of the current study. Despite these limits, this study has the strength to describe a large and real-life cohort of PLWH followed up prospectively in multiple centers across Italy in a research network (SCOLTA), which was specifically designed to improve post-marketing surveillance of adverse reactions to antiretrovirals and with precise expertise in AE monitoring. Moreover, the results were carefully adjusted for confounding factors and seemed robust, due to the sample’s large size.

CNS-AEs leading to ART discontinuation were more frequent in DTG than in non-DTG-treated PLWH. Reassuringly, most AE resolved after ART switch, with similar frequency in DTG and non-DTG cohorts.

## Figures and Tables

**Table 1:** Demographic and clinical characteristics of 4939 study participants on dolutegravir (DTG) or non-DTG containing ART at study entry.

	DTG		Non-DTG		*p*
	*N* ^§^ = 1179	%	*N* ^§^ = 3760	%	
**Sex**					
F	287	24.3	1047	27.8	
M	892	75.7	2713	72.2	0.02
**Age (years)**					
mean ± SD	48.1 ± 12.0		44.2 ± 12.7		<0.0001
≤50	643	54.5	2892	76.9	
>50	536	45.5	868	23.1	<0.0001
**Ethnicity**					
Caucasian	1069	90.7	3469	92.3	
Other	110	9.3	291	7.7	0.08
**Weight (kg)**					
mean ± SD	71.0 ± 13.5		69.0 ± 13.7		<0.0001
**HCV-Ab positive**					
N	889	78.0	2385	65.8	
Y	250	22.0	1238	34.2	<0.0001
**Naïve status**					
N	883	74.9	3222	85.7	
Y	296	25.1	538	14.3	<0.0001
**CDC stage**					
A	603	51.2	1482	39.4	
B	310	26.3	1104	29.4	
C	266	22.6	1174	31.2	<0.0001
**Baseline HIVRNA (experienced)**					
Detectable	146	16.5	1670	51.8	
Undetectable	737	83.5	1552	48.2	<0.0001
**CD4 (cells/mm^3^)**					
median (IQR)	566 (328–798)		371 (197–604)		<0.0001
<250	212	18.0	1233	32.8	
250–499	287	24.3	1233	32.8	
500–749	310	26.3	711	18.9	
≥750	347	29.4	574	15.3	<0.0001
Baseline psychiatric disorder *	84	7.1	235	6.2	0.29

ART: antiretroviral therapy; CDC: Centers for Disease Control and Prevention; DTG: dolutegravir; HCV-Ab: hepatitis C virus Antibody. ^§^ Since not all data were available for the whole study population, the number of available observations is indicated for each study variable. * Major depressive disorder (5.3%), anxiety (0.4%), psychosis (0.6%), schizophrenia (0.3%).

**Table 2:** Frequency of central nervous system (CNS) adverse event (AE) resolution according to patients‘ characteristics and antiretroviral treatment.

	AE Resolved N (%)	AE Not Resolved N (%)	*p*-Value
All (*n* = 63)	58 (92.1)	5 (7.9)	
DTG (*n* = 39)	35 (89.7)	4 (10.3)	0.64
Non-DTG (*n* = 24)	23 (95.8)	1 (4.2)	
INSTI (*n* = 53)	49 (92.9)	4 (7.6)	0.79
Non-INSTI (*n* = 10)	9 (90.0)	1 (10.0)	
Female (*n* = 19)	17 (89.5)	2 (10.5)	
Male (*n* = 44)	41 (93.2)	3 (6.8)	0.63
Age ≤ 50 years (*n* = 34)	34 (100)	0	
Age > 50 years (*n* = 29)	24 (82.8)	5 (17.2)	0.017
Caucasian (*n* = 57)	52 (91.2)	5 (8.8)	
Other (*n* = 6)	6 (100)	0	1.0
HCV-Ab negative (*n* = 40)	35 (87.5)	5 (12.5)	
HCV-Ab positive (*n* = 20)	20 (100)	0 (0.0)	0.16
ART-experienced (*n* = 44)	40 (90.9)	4 (9.1)	
ART-naïve (*n* = 19)	18 (94.7)	1 (5.3)	1.0
CDC stage A (*n* = 30)	28 (93.3)	2 (6.7)	0.95
B (*n* = 17)	15 (88.2)	2 (11.8)	
C (*n* = 16)	15 (93.8)	1 (6.2)	
CD4 < 250 cells/mmc (*n* = 13)	12 (92.3)	1 (7.7)	0.43
250–499 cells/mmc (*n* = 18)	18 (100)	0 (0.0)	
500–749 cells/mmc (*n* = 12)	10 (83.3)	2 (16.7)	
≥750 cells/mmc (*n* = 20)	18 (90.0)	2 (10.0)	
Baseline psychiatric disorder no (*n* = 54)	51 (94.4)	3 (5.6)	
yes (*n* = 9)	7 (77.8)	2 (22.2)	0.14
Switch to INSTI (*n* = 26)	22 (84.6)	4 (15.4)	0.15
Switch to NNRTI (*n* = 14)	13 (92.9)	1 (7.1)	1.0
Switch to PI (*n* = 24)	23 (95.8)	1 (4.2)	0.64
Inter-class switch (*n* = 37) *	36 (97.3)	1 (2.7)	0.15
Intra-class switch (*n* = 26)	22 (88.5)	4 (15.4)	

* this category included two patients who discontinued any ART. ART: antiretroviral therapy; CD4: CD4 + T cell count; DTG dolutegravir; HCV-Ab: hepatitis C virus Antibody; INSTI: integrase inhibitors; NNRTI: non-nucleoside reverse transcriptase inhibitors; PI: protease inhibitors.

**Table 3:** Crude and adjusted hazard ratio (HR) for discontinuation due to central nervous system events.

Variable	Crude HR	95% CI	*p*	Adjusted HR **	95% CI	*p*
Sex F (ref. M)	1.29	0.77–2.17	0.33			
Age (by 1 year)	1.02	1.00–1.04	0.10			
Age (ref. < 50 years)	**1.97**	**1.21–3.21**	**0.006**	1.52	0.91–2.54	0.10
Weight (by 5 Kg)	0.99	0.91–1.08	0.89			
Ethnicity (ref. Caucasian)	1.23	0.53–2.85	0.62			
HCV-Ab+ (ref. HCV-Ab negative)	1.14	0.67–1.92	0.63			
Naïve (ref. experienced)	**2.06**	**1.21–3.51**	**0.008**	**2.23**	**1.24–3.99**	**0.007**
CDC stage (ref. A)						
B	0.88	0.50–1.56	0.66			
C	0.75	0.41–1.36	0.34			
CDC stage: chi-square for trend	0.92		0.34			
CD4 (ref. < 250)						
250–499	1.32	0.65–2.69	0.44	1.32	0.65–2.70	0.44
500–749	1.30	0.59–2.84	0.51	1.11	0.50–2.47	0.80
≥750	**2.65**	**1.34–5.25**	**0.005**	**2.22**	**1.07–4.62**	**0.03**
CD4 class: chi-square for trend	**6.89**		**0.009**	**3.86**		**0.049**
Baseline psychiatric disorder (ref. no) *	**2.35**	**1.16–4.75**	**0.017**	**2.13**	**1.04–4.34**	**0.038**
Non-DTG Cohort (ref. DTG)	**0.25**	**0.15–0.41**	**<0.0001**	**0.33**	**0.19–0.55**	**<0.0001**

* Major depressive disorder, anxiety, psychosis, schizophrenia. ** including variables statistically significant at crude analysis. CDC: Centers for Disease Control and Prevention.

**Table 4:** Crude and adjusted hazard ratio (HR) for discontinuation due to central nervous system events in dolutegravir (DTG) and non-DTG containing regimens.

Variable	Crude HR	95% CI	*p*	Adjusted HR **	95% CI	*p*
**DTG cohort**						
Sex F (ref. M)	1.10	0.54–2.25	0.79			
Age (by 1 year)	1.00	0.97–1.02	0.89			
Age (ref. < 50 years)	1.32	0.71–2.46	0.38			
Weight (by 5 Kg)	0.98	0.88–1.10	0.78			
Ethnicity (ref. Caucasian)	1.28	0.46–3.60	0.64			
HCV–Ab+ (ref. HCV–Ab negative)	1.36	0.66–2.84	0.40			
Naïve (ref. experienced)	**2.53**	**1.35–4.73**	**0.004**			
CDC stage (ref. A)						
B	1.29	0.62–2.71	0.50			
C	1.29	0.59–2.82	0.52			
CDC stage: chi–square for trend	0.53		0.96			
CD4 (ref. < 250)						
250–499	0.72	0.29–1.80	0.48			
500–749	0.53	0.20–1.42	0.21			
≥750	0.97	0.42–2.23	0.94			
CD4 class: chi–square for trend	0.001		0.98			
Baseline psychiatric disorder (ref. no) *	1.05	0.32–3.42	0.93			
**Non–DTG cohort**						
Sex F (ref. M)	1.81	0.84–3.90	0.13			
Age (by 1 year)	1.02	0.89–1.06	0.21			
Age (ref. < 50 years)	1.68	0.75–3.75	0.20			
Weight (by 5 Kg)	0.96	0.84–1.10	0.59			
Ethnicity (ref. Caucasian)	0.99	0.24–4.18	0.99			
HCV–Ab+ (ref. HCV–Ab negative)	1.34	0.62–2.39	0.45			
Naïve (ref. experienced)	0.48	0.11–2.03	0.32			
CDC stage (ref. A)						
B	0.66	0.29–1.63	0.37			
C	0.54	0.21–1.40	0.21			
CDC stage: chi–square for trend	1.72		0.19			
CD4 (ref. < 250)						
250–499	2.42	0.76–7.74	0.14	2.41	0.76–7.68	0.14
500–749	2.17	0.59–8.03	0.25	2.01	0.54–7.51	0.30
≥750	**4.33**	**1.32–14.28**	**0.016**	**4.04**	**1.21–13.44**	**0.02**
CD4 class: chi–square for trend	**5.58**		**0.018**	**4.46**		**0.035**
Baseline psychiatric disorder (ref. no) *	**4.42**	**1.78–10.93**	**0.001**	**4.15**	**1.67–10.33**	**0.002**

* Major depressive disorder, anxiety, psychosis, schizophrenia. ** including variables statistically significant at crude analysis. CDC: Centers for Disease Control and Prevention.

## Data Availability

The dataset used and analyzed during the current study is available from the corresponding author on reasonable request.

## Supplementary Materials

The following supporting information can be downloaded at: https://www.mdpi.com/article/10.3390/v14051028/s1. Figure S1: Survival analysis in strata of integrase inhibitors (INI) and non-INI-based regimens showed a significantly higher rate of discontinuations due to CNS-AE in INI (logrank p < 0.0001). Table S1: Crude and adjusted hazard ratio (HR) for discontinuation due to central nervous system events.
