# Supplementary material for: Reversibility of Central Nervous System Adverse Events in Course of Art
Source: Viruses. 2022 May 11;14(5):1028. doi: 10.3390/v14051028 (PMC9147522; doi:10.3390/v14051028)
Supplement: Supplementary file 1 [file viruses-14-01028-s001.zip › viruses-1665449-supplementary.pdf]

Figure S1*:* Survival analysis in strata of integrase inhibitors (INI) and non-INI-based regimens showed a significantly higher rate of discontinuations due to CNS-AE in INI (logrank *p* <0.0001).

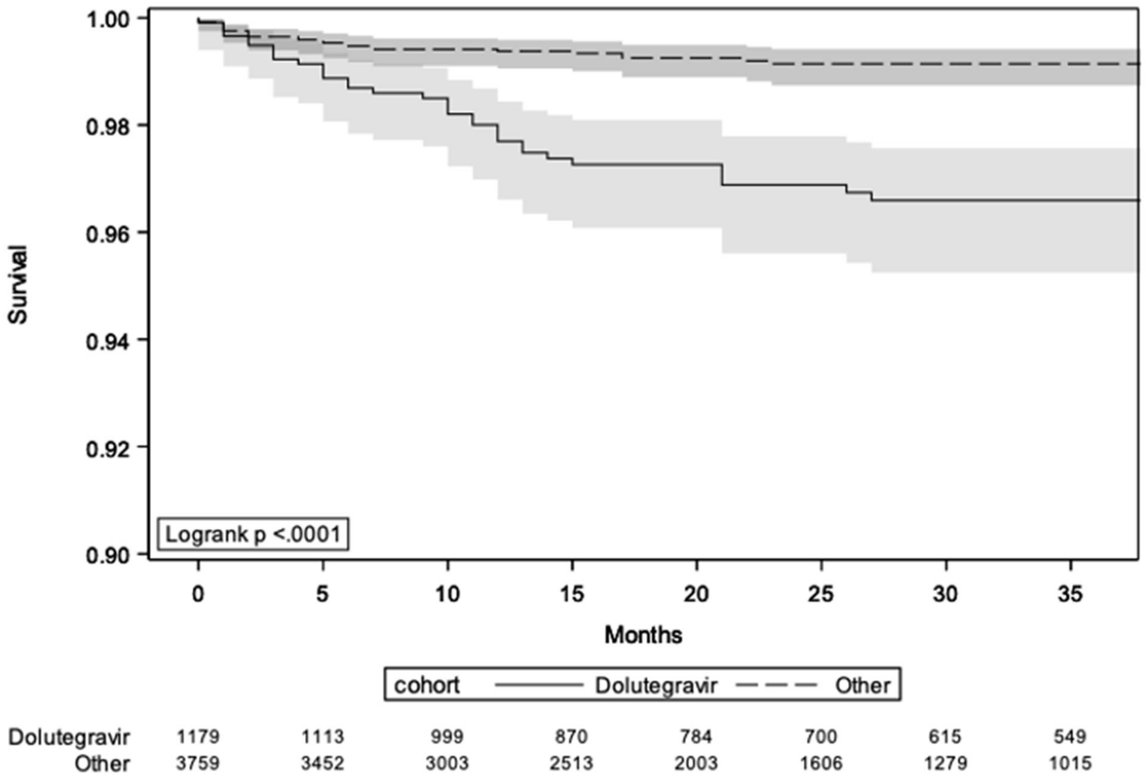

Table S1. Crude and adjusted hazard ratio (HR) for discontinuation due to central nervous system events.

**Variable**	**Crude HR**	**95% CI**	**P**	**Adjusted HR****	**95% CI**	**P**
Sex F (ref. M)	1.28	0.77-2.16	0.34			
Age (by 1 year)	1.02	1.00-1.04	0.10			
Age (ref. ≤50 years)	**1.97**	**1.21-3.21**	**0.006**	1.57	0.94-2.62	0.08
Weight (by 5 Kg)	0.99	0.91-1.08	0.89			
Ethnicity (ref. Caucasian)	1.23	0.53-2.85	0.62			
HCV-Ab+ (ref. HCV-Ab negative)	1.14	0.67-1.92	0.63			
Naïve (ref. experienced)	**2.06**	**1.21-3.51**	**0.008**	**2.44**	**1.39-4.28**	**0.002**
CDC stage (ref. A)						
B	0.88	0.50-1.56	0.66			
C	0.75	0.41-1.36	0.34			
CDC stage: chi-square for trend	0.92		0.34			
CD4 (ref. <250)						
250-499	1.32	0.65-2.69	0.44	1.28	0.64-2.61	0.47
500-749	1.30	0.59-2.84	0.51	1.11	0.49-2.51	0.80
≥750	**2.65**	**1.34-5.25**	**0.005**	**2.28**	**1.08-4.68**	**0.03**
CD4 class: chi-square for trend	**7.02**		**0.008**	3.66		0.055
Baseline psychiatric disorder (ref. no) *	**2.35**	**1.16-4.75**	**0.017**	**2.05**	**1.01-4.18**	**0.049**
Study cohort (ref. Dolutegravir)						
Lopinavir	**0.09**	**0.02-0.40**	**0.0009**	**0.12**	**0.03-0.52**	**0.005**
Atazanavir	**0.06**	**0.01-0.42**	**0.004**	**0.08**	**0.01-0.61**	**0.015**
Darunavir	**0.09**	**0.02-0.37**	**0.0008**	**0.11**	**0.03-0.44**	**0.002**
Rilpivirine	0.63	0.29-1.33	0.22	0.65	0.30-1.43	0.29
Raltegravir	**0.30**	**0.12-0.75**	**0.01**	**0.38**	**0.15-0.98**	**0.045**
Elvitegravir	**0.28**	**0.09-0.89**	**0.03**	**0.29**	**0.09-0.94**	**0.036**
Bictegravir	0.67	0.28-1.59	0.36	0.67	0.28-1.60	0.37
